# Treatment with interleukin‐33 is non‐toxic and protects retinal pigment epithelium in an ageing model of outer retinal degeneration

**DOI:** 10.1111/jcmm.16000

**Published:** 2020-10-20

**Authors:** Alison J. Clare, David A. Copland, Lindsay B. Nicholson, Jian Liu, Chris R. Neal, Stephen Moss, Andrew D. Dick, Sofia Theodoropoulou

**Affiliations:** ^1^ Academic Unit of Ophthalmology Translational Health Sciences University of Bristol Bristol UK; ^2^ School of Cellular and Molecular Medicine University of Bristol Bristol UK; ^3^ Wolfson Bioimaging Facility University of Bristol Bristol UK; ^4^ UCL Institute of Ophthalmology London UK; ^5^ NIHR Biomedical Research Centre of Ophthalmology Moorfields Eye Hospital London UK; ^6^ Department of Ophthalmology Cheltenham General Hospital Cheltenham UK

**Keywords:** age‐related macular degeneration, complement factor H, IL‐33, retinal pigment epithelium

## Abstract

The leading cause of central vision loss, age‐related macular degeneration (AMD), is a degenerative disorder characterized by atrophy of retinal pigment epithelium (RPE) and photoreceptors. For 15% of cases, neovascularization occurs, leading to acute vision loss if left untreated. For the remaining patients, there are currently no treatment options and preventing progressive RPE atrophy remains the main therapeutic goal. Previously, we have shown treatment with interleukin‐33 can reduce choroidal neovascularization and attenuate tissue remodelling. Here, we investigate IL‐33 delivery in aged, high‐fat diet (HFD) fed mice on a wildtype and complement factor H heterozygous knockout background. We characterize the non‐toxic effect following intravitreal injection of IL‐33 and further demonstrate protective effects against RPE cell death with evidence of maintaining metabolic retinal homeostasis of *Cfh*+/−~HFD mice. Our results further support the potential utility of IL‐33 to prevent AMD progression.

## 1 INTRODUCTION

Age‐related macular degeneration (AMD) is a degenerative disease of the eye and the leading cause of central vision loss. Characterized by drusen deposits, atrophy of the retinal pigment epithelium (RPE) and photoreceptor (PR) loss, AMD can progress into two stages of vision loss, acute neovascularization AMD (nAMD) and, in the majority of patients, pernicious geographic atrophy (aAMD). Whilst nAMD can effectively be treated in most patients with VEGF‐blocking agents, aAMD currently has no therapeutic options.^1^, ^2^

Dysregulation of the immune system homeostasis is operative in the pathogenesis of AMD.^2^ We previously revealed a protective role for interleukin‐33 (IL‐33), in choroidal neovascularization and attenuation of wound healing.^3^ IL‐33 is constitutively expressed in the RPE and Müller cells and acts as an ‘alarmin’ under necrotic cell death.^3^, ^4^ Loss of endogenous IL‐33 leads to chronic inflammation after retinal detachment.^5^ Conversely, under acute stress, endogenous IL‐33 released from Müller cells triggers an inflammatory response and PR degeneration.^4^ The divergence in data needs to be reconciled to understand whether exogenous IL‐33 intervention early in disease could promote homeostasis and repair.

In this study, we use an insidious aAMD model, aged heterozygous complement factor H knockout (*Cfh*+/−) mice on a high‐fat diet (HFD)^6^ to determine the safety of IL‐33 treatment and demonstrate its potential as an early therapeutic for preventing AMD progression.

## 2 METHODS

### 2.1 Mice and in vivo experimental procedures

C57BL/6J mice (rd8^−/−^) were acquired from Charles River Laboratories (Margate, UK). *Cfh*−/− mice were backcrossed to C57BL/6J mice, establishing *Cfh*+/− mice within the University of Bristol Animal services Unit. Procedures were performed according to University of Bristol institutional guidelines approved under Home Office Project License 30/3281, and the Association for Research in Vision and Ophthalmology (ARVO) statement.

Aged mice (80‐99 weeks) were switched from normal diet to HFD (Envigo), for eight weeks.^6^ Administration of recombinant mouse IL‐33 (ALX‐522‐101‐C010, Enzo Life Sciences Ltd) or, in contralateral eye, vehicle control (RPMI, 5% foetal calf serum; 0.5% penicillin/streptomyocin; 0.5% L‐glutamine; 0.5 mmol/L Sodium Pyruvate, 2.5 ng/mL recombinant murine IL‐3 [Thermo Fisher Scientific] and 5 ng/mL recombinant murine SCF [Stem Cell Technologies]) was delivered by 2 µL intravitreal injection. In vivo imaging optical coherence tomography (OCT) scans of retina were captured using Micron IV (Phoenix Research Laboratories). Retinal thickness was measured using FIJI.^7^

### 2.2 Whole‐mount immunohistochemistry and microscopy

For whole‐mounts, posterior eye cups were dissected and fixed in 2% v/v formaldehyde overnight at 4°C. Retina and RPE/choroid were blocked in 5% w/v bovine serum albumin with 0.3% v/v Triton X‐100 (Sigma Aldrich) in PBS before staining with TMR Red‐dUTP TUNEL (Roche Diagnostics), followed by incubation with ZO‐1 antibody (1 in 50; 40‐2200; Thermo Fisher Scientific) overnight at 4°C. Tissue was then incubated with appropriate secondary antibody (A‐11034; Invitrogen; 1 in 200) and mounted in hard‐set medium (Vector Laboratories). Images were captured using a Leica SP5‐AOBS confocal laser scanning microscope (Leica Microsystems Ltd.) and TUNEL spots identified using Volocity^®^ Image Analysis Software 6.0. Data are presented as mean number of TUNEL + cells per field of view (FOV).

### 2.3 Electron microscopy

For transmission electron microscopy (TEM), eyes were fixed in 2.5% v/v glutaraldehyde initially at RT for 2 hours, before further fixing of posterior cup only at 4°C until processed. Eye cups were fixed in osmium tetroxide, stained with uranyl acetate and dehydrated through a graded ethanol series to 100% ethanol, before incubation with propylene oxide and infiltration with Epon resin. Eyes were then embedded and polymerized at 60°C for 2 days. Thin sections (70‐80 nm) were prepared and stained with lead and uranyl salts. Images were captured using a Tecnai T12 microscope (Thermo Fisher Scientific). Across four sections, 55‐60 images were captured per eye and fifteen measurements/image were made along the Bruch's membrane using FIJI.^7^

### 2.4 Protein lysate and Western blots

Whole retinas were crushed in 200 µL Pierce^®^ RIPA buffer (Thermo Fisher Scientific) with protease inhibitor (Cell Signalling Technologies) and samples were separated by SDS‐PAGE before transfer to PVDF. Following blocking with 5% w/v milk in TBS + Tween 20 (0.1% v/v), blots were incubated with primary antibodies (all antibodies Cell signalling Technologies) Hexokinase II (2867; 1 in 1000) and β‐actin (3700; 1 in 1000) at 4°C overnight. Protein was detected with HRP‐conjugated secondary antibody, anti‐rabbit (1 in 2000) or anti‐mouse (1 in 2000) and chemiluminescent (GE Healthcare). Densitometry was performed using FIJI.^7^

### 2.5 Statistics

Analysis of data used three‐way ANOVA in R studio (R version 3.6.1; RStudio, Inc) with independent factors, treatment, genotype and animal (accounting for paired eyes). A Tukey multiple comparisons test was used where significant effects were found. If no significance was identified from further multiple comparisons, the result of three‐way ANOVA is presented to demonstrate significant effect of independent factor. Mean sub‐RPE deposits were analysed using a paired t test in GraphPad Prism (version 8.1.2; GraphPad Software Inc). All data are expressed as ± SEM.

## 3 RESULTS AND DISCUSSION

**Figure 1:**
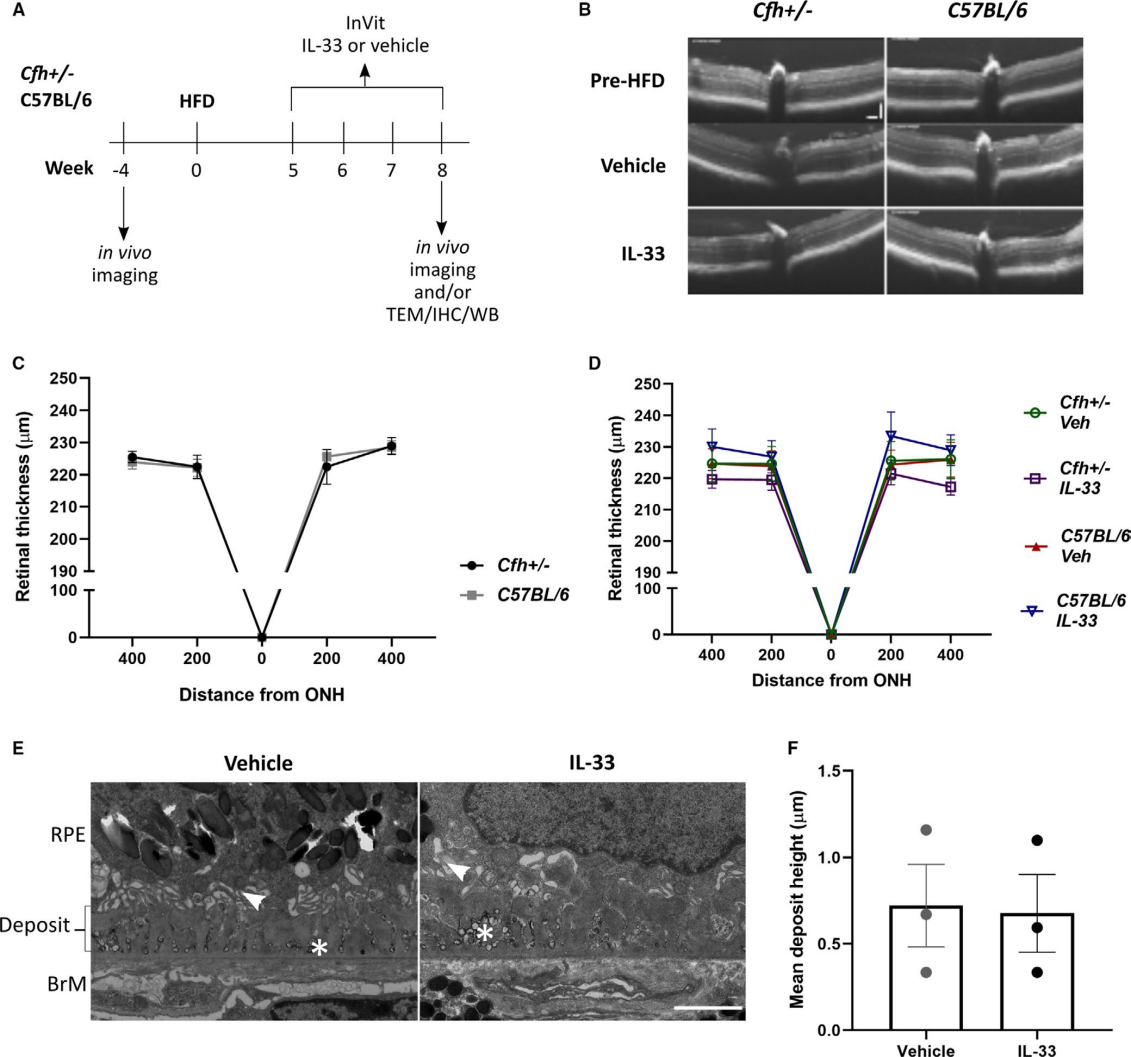
Exogenous IL‐33 is non‐toxic in aged C57BL/6 ~ HFD and *Cfh*+/−~HFD mice. (A) Schematic to explain timeline of animal manipulations; TEM—transmission electron microscopy, IHC—immunohistochemistry, WB—Western blot (B) OCT scan of eyes from *Cfh ± *and C57BL/6 mice prior to high‐fat diet (HFD) and post‐HFD animals injected with either vehicle or IL‐33 (1 ng/µL). Scale bars are both 100 µm. Graph to show retinal thickness measured from OCT scans of *Cfh±* (n = 4) and C57BL/6 mice prior to high‐fat diet start (n = 6) (C) or *Cfh*+/−~HFD or C57BL/6 ~ HFD mice injected with either IL‐33 or vehicle control (n = 4‐11) (D). (E) Transmission electron micrograph images of sub‐RPE deposit in *Cfh*+/−~HFD mice treated with either IL‐33 (1 ng/µL) or vehicle. Arrowhead shows remnants of basal infoldings disrupted by deposit formation. In both IL‐33 and vehicle‐treated eyes, large numbers of vesicle‐like structures with an electron‐dense shell are observed within the deposits, as indicated by the asterix (*). BrM—Bruch's membrane. Scale bar is 2 µm. (F) Graph shows mean deposit height in eyes of *Cfh*+/−~HFD IL‐33 and vehicle‐treated eyes (n = 3 of each)

The *Cfh*+/−~HFD model presents with distinct disease phenotype, without significant cell loss,^6^ providing opportunity to measure treatment toxicity and efficacy in a mimic of early/intermediate aAMD. For analysis of IL‐33 treatment in this model, we first assessed a non‐toxic dose, based on efficacious treatment doses.^3^ Animals received a weekly delivery of 1 ng/µL IL‐33 or vehicle control, from the fifth week of HFD (Figure 1A). With acute models of retinal degeneration, release of endogenous IL‐33 leads to significant retinal thinning.^4^ Here, retinal thickness at week 8 was unaffected by IL‐33 treatment in control C57BL/6 ~ HFD and *Cfh*+/−~HFD animals (Figure 1B,D), with thickness remaining similar to eyes pre‐manipulations (Figure 1A‐C).

We further determined IL‐33 toxicity by TEM analysis of sub‐RPE deposits, a key pathological feature in the *Cfh*+/−~HFD model.^6^ Here, we observed deposits in vehicle and IL‐33‐treated *Cfh*+/−~HFD eyes (Figure 1E), with no difference in mean height (n = 3, 3, Figure 1F). Deposit formation in AMD is considered to be driven by a dysregulated immune response and local inflammation.^8^ Toxicity was not apparent as no exacerbation of pathological deposit formation was observed with IL‐33 treatment.

**Figure 2:**
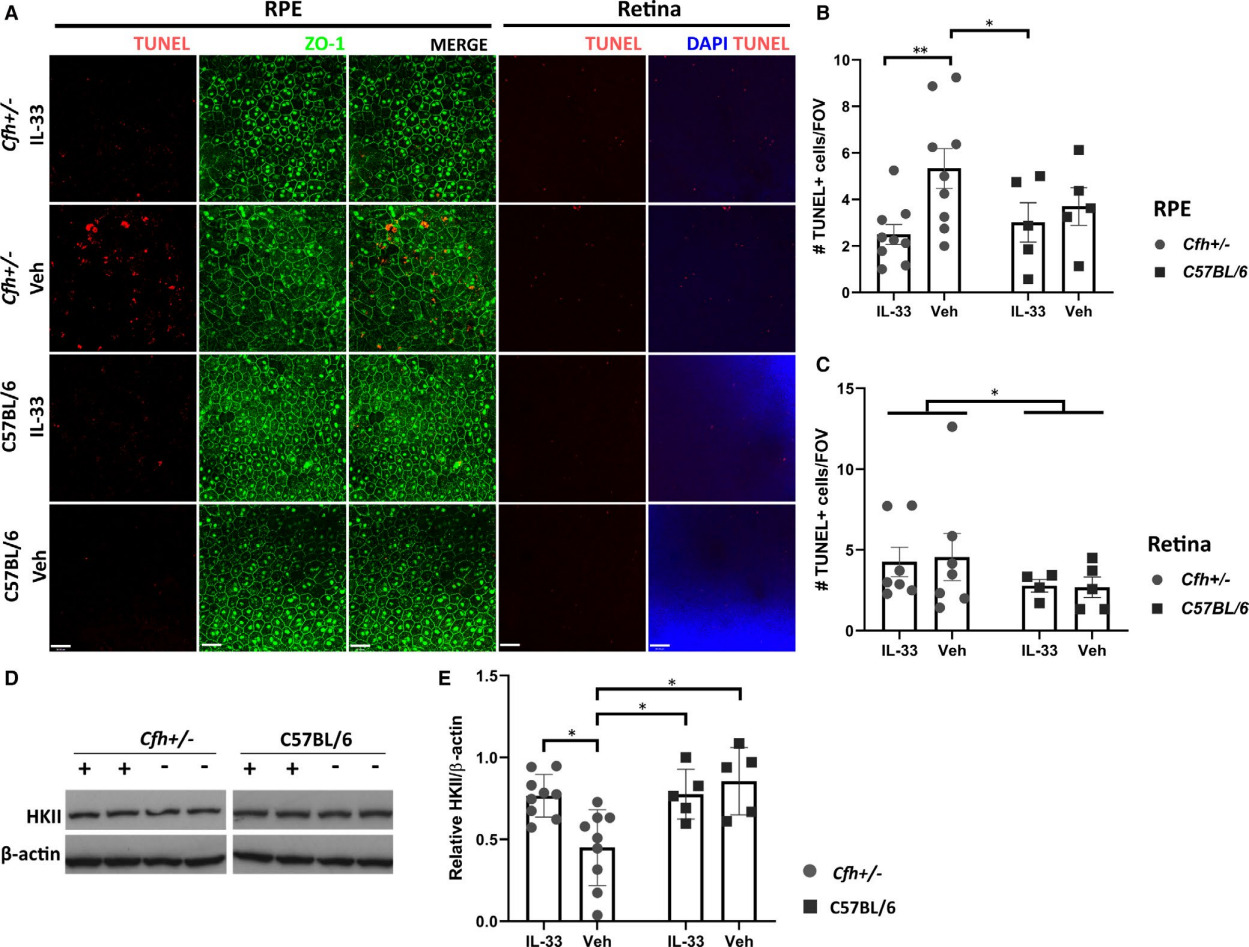
IL‐33 treatment protects against RPE cell death and loss of metabolically important protein Hexokinase II in retina in *Cfh*+/−~HFD mice. A, Confocal images of ZO‐1/TUNEL stained RPE/choroid and DAPI/TUNEL stained retina flatmounts from *Cfh*+/−~HFD and C57BL/6 ~ HFD eyes treated with either vehicle or IL‐33 (1 ng/µL). Scale bar is 38 µm. B, Quantitative analysis of mean TUNEL + cells per field of view in RPE/choroid flatmounts. A significant reduction in cell death is observed in *Cfh*+/−~HFD IL‐33‐treated eyes (n = 9) compared to *Cfh*+/−~HFD vehicle‐treated eyes (n = 9; **P = .0037) and C57BL/6 ~ HFD IL‐33‐treated eyes (n = 5; *P = .040). C, Quantitative analysis of TUNEL + cells per field of view in retina. Cell death was significantly increased in *Cfh*+/−~HFD eyes (n = 7, 7) compared to C57BL/6 ~ HFD control (n = 4, 5), irrespective of treatment (*P = .032). D, Representative Western blot of Hexokinase II (HKII) protein expression in retina lysates, cropped lanes from the same blot. E, Densiometric analysis demonstrates HKII is significantly reduced in *Cfh*+/−~HFD vehicle‐treated eyes (n = 9) compared to *Cfh*+/−~HFD IL‐33 (1 ng/µL; n = 9), C57BL/6 ~ HFD veh (n = 5) and IL‐33 (1 ng/µL; n = 5)‐treated eyes (*P = .017, *P* = .010 and *P* = .039, respectively)

Sub‐RPE deposits in *Cfh*+/−~HFD mice are thought to drive complement‐mediated monocytosis and cytotoxicity, resulting in subsequent RPE damage and atrophy.^6^ Indeed, activated monocytes and pro‐inflammatory cytokines can induce reactive oxygen species (ROS) production and cell death in RPE.^9^ Multiple comparisons did not reveal significant difference in extent of cell death between C57BL/6 ~ HFD vehicle eyes and any other group. Notwithstanding, there was a significant increase in cell death in the RPE of vehicle‐treated *Cfh*+/−~HFD eyes, compared to C57BL/6 ~ HFD IL‐33‐treated eyes (Figure 2A‐B). The rescue of cell death in *Cfh*+/−~HFD animals with IL‐33 was to levels found in C57BL/6 ~ HFD eyes (Figure 2B). As IL‐33 treatment did not affect deposit formation, it supports protection of RPE is through re‐balancing of any pro‐inflammatory responses or protection from ROS. Certainly, IL‐33 promotes a skew to a protective non‐inflammatory type 2 immune response in EAU and CNS injury.^10^, ^11^ Additionally, our previous work has found IL‐33 signalling via ST2 promotes normal bioenergetic source of oxidative phosphorylation (OXPHOS) in RPE, protecting from oxidative stress. TLR‐stimulation of RPE induces a switch to glycolysis, essential to subsequent pro‐inflammatory cytokine release.^12^ IL‐33 promotion of RPE metabolic homeostasis would negate any pro‐inflammatory effect during stress conditions, protecting RPE from cell death.

Another indicator of disease is loss of photoreceptors. Analysis of cell death in retina flatmounts revealed a significant genotype and model effect with higher TUNEL‐positive cells in all *Cfh*+/−~HFD animals compared to all C57BL/6 ~ HFD. No treatment effect was observed (Figure 2A,C). In aAMD, loss of RPE precludes significant PR loss.^13^ Whilst there is no effect of IL‐33 treatment on cell loss in the retina early in the course, the RPE protection conferred is predicted to prevent cell loss with time.

To further investigate the potential impact for IL‐33 protection of RPE on retinal health and support the metabolic regulatory role,^12^ we employed Hexokinase II (HKII). The energetically demanding retina relies on aerobic glycolysis, for which HKII is an essential mediator.^14^ Conversely, RPE utilizes lactate for OXPHOS; however, in the context of mitochondrial oxidative stress, RPE can become more glycolytic, starving the retina of its energy source.^15^ Here, in *Cfh*+/−~HFD vehicle control retina lysates we observed significant loss of HKII compared to C57BL/6 ~ HFD vehicle and C57BL/6 ~ HFD IL‐33‐treated retinas (Figure 2D; Supplementary Information). IL‐33 treatment in *Cfh*+/−~HFD retinas significantly recovered HKII expression to levels similar in C57BL/6 eyes (Figure 2E), supporting IL‐33 treatment maintenance of metabolic homeostasis.

Our results demonstrate a non‐toxic effect for exogenous IL‐33 treatment in aged mice. Moreover, we reveal a protective capability of IL‐33 against RPE loss and for retina metabolic homeostasis in a dysregulated immune‐mediated insidious model of outer retinal degeneration (*Cfh*+/−~HFD animals). Together, these data highlight the potential of IL‐33 treatment to protect during early pathogenesis of AMD.

## CONFLICT OF INTEREST

The authors declare no competing interests.

## AUTHOR CONTRIBUTION

**Alison Jane Clare:** Data curation (lead); Formal analysis (lead); Investigation (lead); Methodology (lead); Writing‐original draft (lead); Writing‐review & editing (equal). **Dave A Copland:** Conceptualization (equal); Data curation (supporting); Formal analysis (supporting); Investigation (supporting); Methodology (equal); Project administration (equal); Writing‐review & editing (equal). **Lindsay B Nicholson:** Conceptualization (equal); Project administration (supporting); Writing‐review & editing (equal). **Jian Liu:** Formal analysis (supporting); Methodology (supporting); Writing‐review & editing (equal). **Chris Neal:** Formal analysis (supporting); Methodology (supporting); Writing‐review & editing (equal). **Dr Stephen E Moss:** Resources (equal); Writing‐review & editing (equal). **Andrew D Dick:** Conceptualization (lead); Formal analysis (supporting); Project administration (equal); Supervision (equal); Writing‐original draft (equal); Writing‐review & editing (equal). **Sofia Theodoropoulou:** Conceptualization (lead); Formal analysis (equal); Funding acquisition (equal); Project administration (lead); Supervision (lead); Writing‐review & editing (equal).

## Supporting information

Supplementary Material

## Data Availability

The datasets generated and/or analysed during the current study are available from the corresponding author on reasonable request.
