# Supplementary material for: Treatment with interleukin‐33 is non‐toxic and protects retinal pigment epithelium in an ageing model of outer retinal degeneration
Source: J Cell Mol Med. 2020 Oct 20;24(22):13546–50. doi: 10.1111/jcmm.16000 (PMC7701527; doi:10.1111/jcmm.16000)
Supplement: Supplementary file 1 — Supplementary Material [file JCMM-24-13546-s001.docx]

**Title: Treatment with interleukin-33 is non-toxic and protects retinal pigment epithelium in an ageing model of outer retinal degeneration**

Alison J. Clare^1*^, David A. Copland^1^, Lindsay B. Nicholson^1,2^, Jian Liu^1^, Chris R. Neal^3^, Stephen Moss^5^, Andrew D. Dick^1,2,4,5^, Sofia Theodoropoulou^1,6^

^1^Academic Unit of Ophthalmology, Translational Health Sciences, University of Bristol, Bristol, UK

^2^School of Cellular and Molecular Medicine, University of Bristol, Bristol, UK

^3^Wolfson Bioimaging Facility, University of Bristol, UK

^4^NIHR Biomedical Research Centre of Ophthalmology, Moorfields Eye Hospital, London, UK

^5^UCL Institute of Ophthalmology, London, UK

^6^Department of Ophthalmology, Cheltenham General Hospital, Cheltenham, UK

*Corresponding author: Alison J. Clare, Academic Unit of Ophthalmology, Translational Health Sciences, University of Bristol, Bristol, UK. Email: alison.clare@bristol.ac.uk


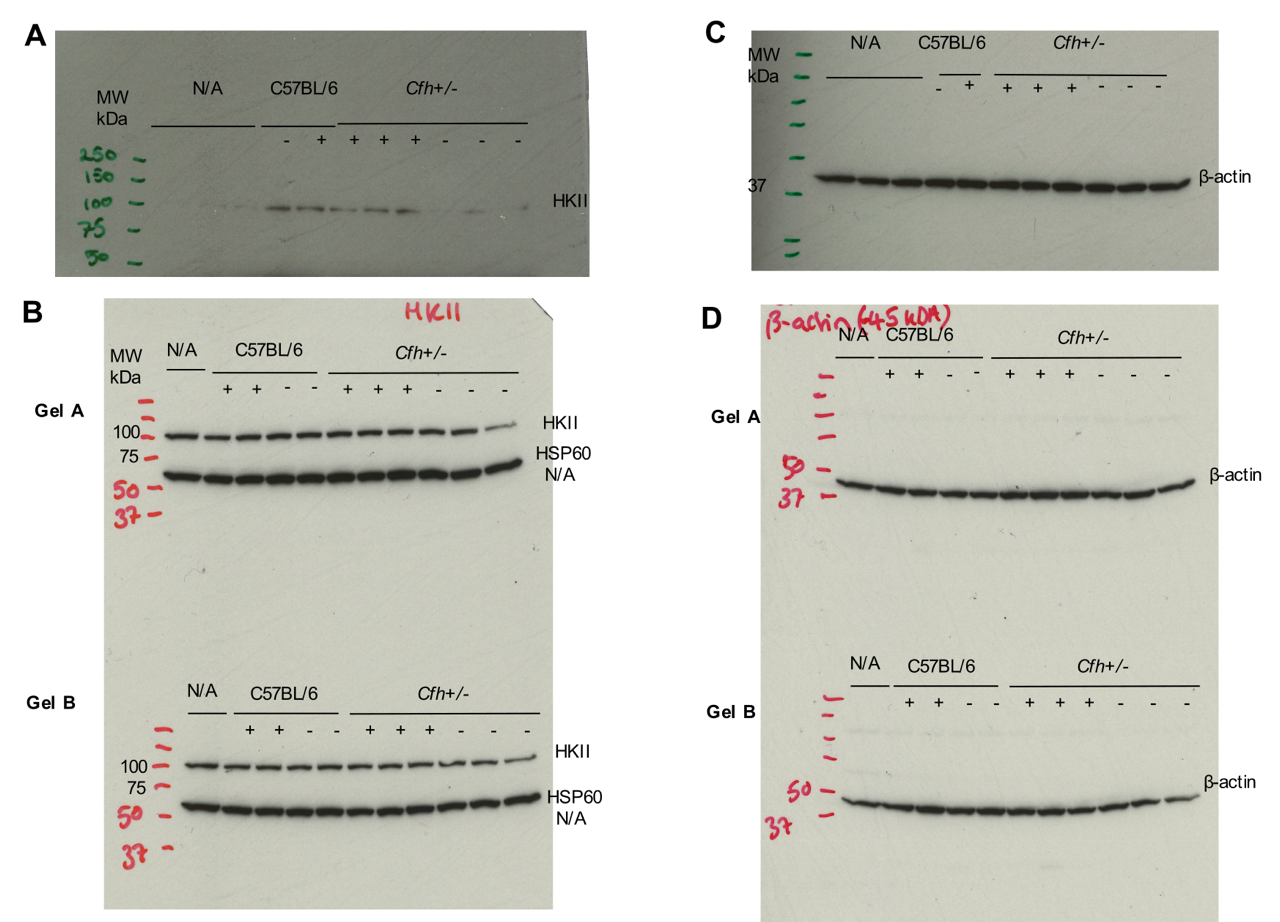


**Supplementary Information.** Original scans of retina samples for analysis of hexokinase II (HKII) expression (102 kDa) **(A)** and **(B)**, relative to β-actin (45 kDa) **(E)** and **(F)**. Samples analysed were split over three gels, with C57BL/6 controls and *Cfh+/-* animals split evenly across gels. Samples run on the same gel not applicable to this study are denoted N/A. Additional bands seen on blot in **(B)** are from earlier probing, the proteins probed for are noted and highlighted as not applicable (N/A) for that blot.
